# FfCOX17 is Involved in Fumonisins Production, Growth, Asexual Reproduction, and Fungicide Sensitivity in *Fusarium fujikuroi*

**DOI:** 10.3390/toxins14070427

**Published:** 2022-06-22

**Authors:** Xuewei Mao, Zhiwen Wu, Furong Chen, Mingguo Zhou, Yiping Hou

**Affiliations:** College of Plant Protection, Nanjing Agricultural University, Nanjing 210095, China; 2018202062@njau.edu.cn (X.M.); 2020202063@stu.njau.edu.cn (Z.W.); 2021202063@stu.njau.edu.cn (F.C.)

**Keywords:** *Fusarium fujikuroi*, *FfCOX17*, vegetative growth, fumonisins, fungicide sensitivity

## Abstract

*Fusarium fujikuroi*, a causal agent of Rice Bakanae Disease, produces secondary metabolites such as gibberellin, pigments bikaverin, and mycotoxins fumonisins. Fumonisins produced by *F. fujikuroi* pose a severe threat to human and animal health. The copper chaperone protein plays a critical role in different growth stages of plants, fungi, and yeasts, but their functions and regulation in fumonisin biosynthesis are still unclear. Here, a copper chaperone protein, *FfCOX17*, was identified in *F. fujikuroi*. The *FfCOX17* deletion mutant (*∆FfCOX17*) exhibited decreased vegetative growth and asexual reproduction. The transcriptional level of the *FfFUM2* gene was significantly induced in *∆FfCOX17*, and the fumonisin production in *∆FfCOX17* mutants was significantly increased compared to wild-type *F. fujikuroi*, but the pathogenicity of *∆FfCOX17* mutants was unaffected, which may be caused by the no significantly changed gibberellin content. *∆FfCOX17* showed decreased sensitivity to oxidative stress, osmotic stress, and increased sensitivity to cell wall stress, heat shock stress, and high concentration glucose. In addition, *∆FfCOX17* also showed increased sensitivity to fungicide fluazinam and fludioxonil, and decreased sensitivity to phenamacril and prochloraz. Taken together, this study suggested that *FfCOX17* is critical for fumonisin production, vegetative growth, asexual reproduction, and fungicide sensitivity, but is not required for the virulence function of *F. fujikuroi* on rice.

## 1. Introduction

*Fusarium fujikuroi* Nirenberg (teleomorph: *Gibberella fujikuroi* mating population C) belongs to the *G. fujikuroi* (Sawada) Wollenweber species complex [1] and is an important pathogenic fungus of Rice Bakanae Disease (RBD) [2]. RBD leads to abnormal growth of rice, yellowing of leaves, reduction of tillers, and empty grains of rice. The majority of these symptoms are caused by gibberellin (GAs), which is a plant hormone [3,4]. In addition to GAs, *F. fujikuroi* produces other secondary metabolites, including mycotoxin fumonisins (FUM), fusarins (FUS), fusaric acid (FU), pigment bikaverin (BIK), and apicidin F (APF), which seriously threaten the yield and quality of rice [5,6,7]. Fumonisins are currently considered one of the most important fungal toxins in agriculture, as they are not only responsible for animal diseases, but are also associated with some human disease epidemiology [8,9,10]. Fumonisins are polyketide-derived metabolites that can inhibit ceramide synthase, a key enzyme in sphingolipid metabolism, and induce apoptosis [8,10]. The fumonisin biosynthetic genes are clustered in *F. fujikuroi*, including 16 *FUM* genes [11]. FUM synthesis appears to be regulated by various environmental factors, such as pH and nutrient sources, at the transcriptional level [12,13]. A previous study has shown that the mitogen-activated protein kinase (MAPK) signaling pathway is involved in the regulation of FUM production in *F. verticillioides* [14]. Carbon sources have also been shown to regulate FUM biosynthesis in *F. proliferatum* [15]. Recent studies suggested that post-translational modifications play a key role in the production of FUM in *F. verticillioides* [16]. So far, the molecular mechanisms underlying FUM regulation have not yet been elucidated. 

The copper chaperone protein COX17 is a factor that promotes the binding of copper to cytochrome c oxidase (CcO) and can transfer heavy metals from the cytoplasm to the mitochondrial membrane space [17]. In *S. cerevisiae*, the mutation of *COX17* causes special defects of cytochrome c oxidase, resulting in respiratory deficiency [18]. Mammalian copper chaperone Cox17p has an essential role in the activation of cytochrome c oxidase and embryonic development [19,20,21]. In *Arabidopsis*, the deletion or mutation of *COX17* results in a deficiency in cell growth and stress response [22,23]. The deletion mutant of the *COX17* homologous gene in the fungus *A. fumigatus*, *ΔAfCOX17*, shows significant defects in mycelial growth [24]. However, the function and regulation of *COX17* in *F. fujikuroi* remain unknown. 

In this study, *FfCOX17*, encoding a cytochrome c oxidase copper chaperone, was identified in *F. fujikuroi*. We confirmed that *FfCOX17* plays a vital role in FUM production, *FfFUM2* gene expression, vegetative growth, asexual development, stress responses, and sensitivity to fungicides. These findings will provide a basis for exploring the regulation of *FfCOX17* on mycotoxins FUM production in *F. fujikuroi*.

## 2. Results

### 2.1. Identification, Deletion, and Complementation of FfCOX17

**Figure 1:**
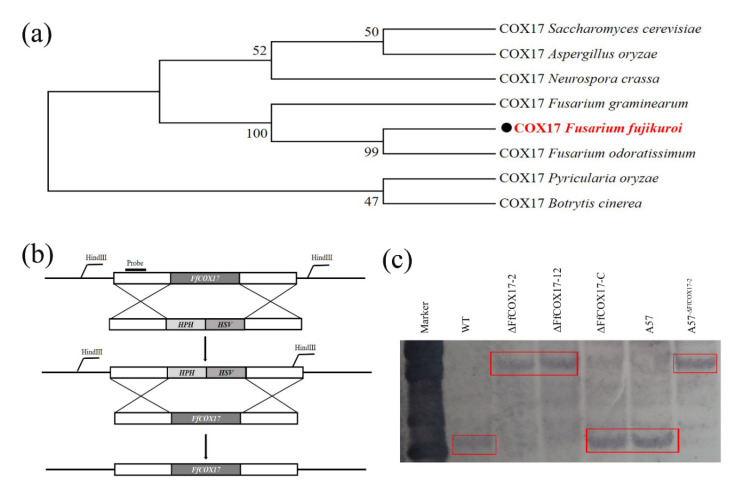
Identification, deletion, and complementation of *FfCOX17*; (**a**) Phylogenetic analysis of COX17 from *S. cerevisiae*, *A. oryzae*, *N. crasse*, *F. graminearun*, *F. fujikuroi*, *F. odoratissimum*, *P. oryzae*, and *B. cinerea*. (**b**) The *ΔFfCOX17* knockout vector was constructed via a homologous replacement strategy. (**c**) Southern blotting analysis of wild-type strain WT, *ΔFfCOX17*-2, *ΔFfCOX17*-12, and *ΔFfCOX17*-C using a 500 bp *FfCOX17* upstream fragment as a probe, and genomic DNA digested with HindIII.

The cytochrome c oxidase copper chaperone *FfCOX17* (FFUJ_01072) was identified from the *F. fujikuroi* genome database (http://fungi.ensembl.org/Fusarium_fujikuroi_gca_001023065/Info/Index (accessed on 13 July 2020) by BLASTP using the *S. cerevisiae* COX17 as a query. *FfCOX17* is a 348 bp gene with two introns and three exons and encodes a protein with seventy-six amino acids. Phylogenetic tree analysis showed that *FfCOX17* was relatively conserved in *Fusarium* spp and other species (Figure 1a). To investigate the role of the *FfCOX17* in *F. fujikuroi*, two independent *FfCOX17* deletion mutants (*ΔFfCOX17-2* and *ΔFfCOX17-12*) were obtained by homologous recombination (Figure 1b), and these mutants were verified by PCR and further confirmed by Southern blotting (Figure 1c). To confirm whether the observed phenotypes of *ΔFfCOX17* were caused by knockout, the complemented mutant (*ΔFfCOX17-C*) was generated by transformation. 

### 2.2. FfCOX17 Is Involved in Vegetative Growth and Asexual Reproduction

**Figure 2:**
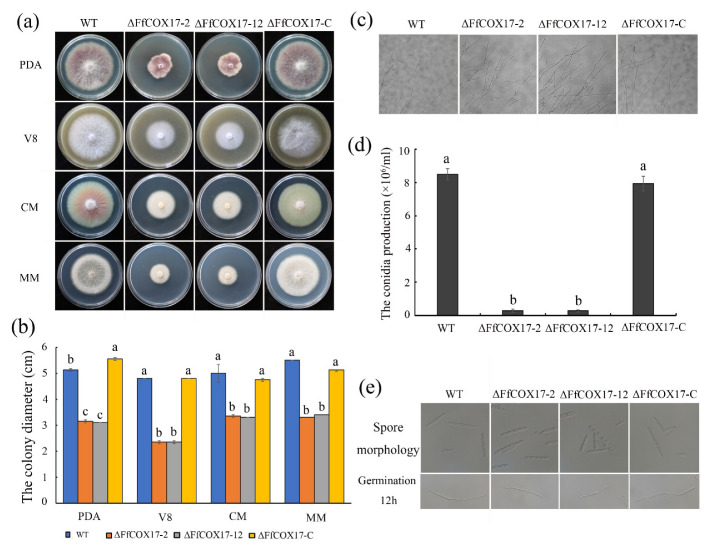
The effects of *FfCOX17* on vegetative growth and asexual reproduction; (**a**) Colony morphology of the wild-type strain WT and the mutant strains of *FfCOX17* on PDA, V8, CM, and MM medium at 25 °C for 7 days. WT: the wild-type strain; *ΔFfCOX17*-2 and *ΔFfCOX17*-12: the deletion mutant of *FfCOX17*; *ΔFfCOX17*-C: complemented strains of *FfCOX17* deletion mutant; (**b**) The diameter of mycelial growth of wild-type strain WT, *ΔFfCOX17*-2, and *ΔFfCOX17*-12 on PDA, V8, CM and MM media at 25 °C for 7 days. (**c**) The hyphal tip morphology of the mycelium of wild-type strain WT, *ΔFfCOX17*-2, *ΔFfCOX17*-12, and complement strain. (**d**) The conidia production of wild-type strain WT, *ΔFfCOX17-2*, *ΔFfCOX17*-12, and complement strain cultured in CMC liquid medium for 7 days. (**e**) The spore morphology and germination of WT, *ΔFfCOX17*-2, *ΔFfCOX17*-12, and complement strain were cultured in CMC liquid medium. Values in each column with the same letter are not significantly different (Tukey; *p* = 0.05).

The deletion mutants of *FfCOX17* were used to confirm the function of *FfCOX17* in *F. fujikuroi*. The growth rate of *ΔFfCOX17-2* and *ΔFfCOX17-12* on PDA, V8, CM, and MM medium was significantly lower than that of the wild-type strain (Figure 2a,b, Table 1). The growth defect of *ΔFfCOX17* was restored in the complement strain *ΔFfCOX17-C*, indicating that *FfCOX17* is involved in vegetative growth in *F. fujikuroi*. Microscopic examination showed that the hyphal tips of *ΔFfCOX17* were dense, and the apical branches of hyphae increased compared with the wild-type strain (Figure 2c).

In *ΔFfCOX17* mutants, the number of conidia was significantly decreased, and the conidia were not typical sickle-shaped (Figure 2d,e, Table 1). The germ tube length at 12 h after germination was shorter compared with wild-type strains (Figure 2e), which suggested that *FfCOX17* is required for asexual reproduction.

### 2.3. FfCOX17 Regulates the Expression Level of the BIK Synthesis-Related Genes

**Figure 3:**
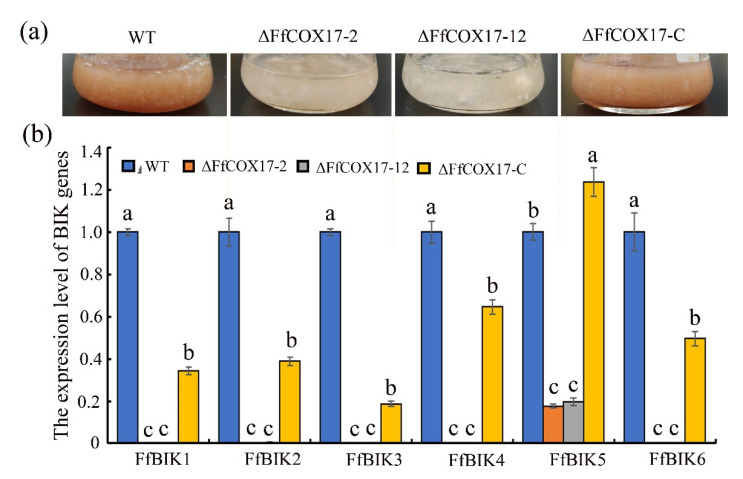
The effects of *FfCOX17* on the expression level of BIK biosynthesis-related genes; (**a**) the pigment changes of wild-type, *ΔFfCOX17*-2, *ΔFfCOX17*-12 and complement strain in ICI liquid medium at 25 °C for 7 days (175 rpm). (**b**) The relative expression levels of BIK genes in the mutant strains of *ΔFfCOX17* on ICI liquid medium at 25 °C for 48 h (175 rpm). *FfBIK1* (FFUJ_06742), *FfBIK2* (FFUJ_06743), *FfBIK3* (FFUJ_06744), *FfBIK4* (FFUJ_06745), *FfBIK5* (FFUJ_06746), *FfBIK6* (FFUJ_06747). Values in each column with the same letter are not significantly different (Tukey; *p* = 0.05).

As shown in Figure 3a, the *∆FfCOX17* mutants were not able to produce pigment when cultured in ICI liquid medium (containing 6 mM Gln), and the expression levels of all six BIK biosynthetic genes were downregulated in the *∆FfCOX17* mutants (Figure 3b). Interestingly, we found that the expression of *FfBIK1*, *FfBIK2*, *FfBIK3*, *FfBIK4*, and *FfBIK6* genes are rarely detected in *∆FfCOX17* mutants, and the expression level of *FfBIK5* decreased by about 80% (Figure 3b), which suggested that *FfCOX17* positively regulates the expression of BIK synthesis related genes in *F. fujikuroi*. 

### 2.4. FfCOX17 Negatively Regulates FUM Biosynthesis in F. fujikuroi

**Figure 4:**
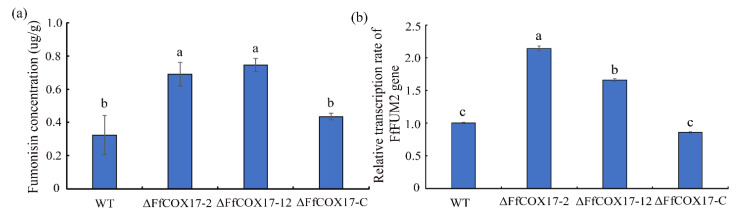
The effects of *FfCOX17* on FUM production. (**a**) The content of FUM of wild-type strain, *ΔFfCOX17* deletion mutants, and complement strain. (**b**) The expression levels of the *FfFUM* gene (FUM2, FFUJ_09248) in wild-type strain WT, *ΔFfCOX17*-2, *ΔFfCOX17*-12, and complement strain cultured in ICI liquid medium for 48 h. Values in each column with the same letter are not significantly different (Tukey; *p* = 0.05).

To determine the role of *FfCOX17* in *F. fujikuroi* FUM biosynthesis, the FUM content was measured in the wild-type, *ΔFfCOX17* mutants, and complemented strain. *ΔFfCOX17* mutants exhibited a significant increase in FUM production compared to that in wild-type and complemented strain (Figure 4a). Next, we quantified the transcriptional changes of *FfFUM2* (FFUJ_09248) gene. Figure 4b showed that the expression levels of the *FfFUM2* gene in *∆FfCOX17* mutants were significantly increased, suggesting that *FfCOX17* controls the FUM production by regulating the transcription level of the *FfFUM2* gene.

### 2.5. FfCOX17 Is Not Required for Pathogenicity

**Figure 5:**
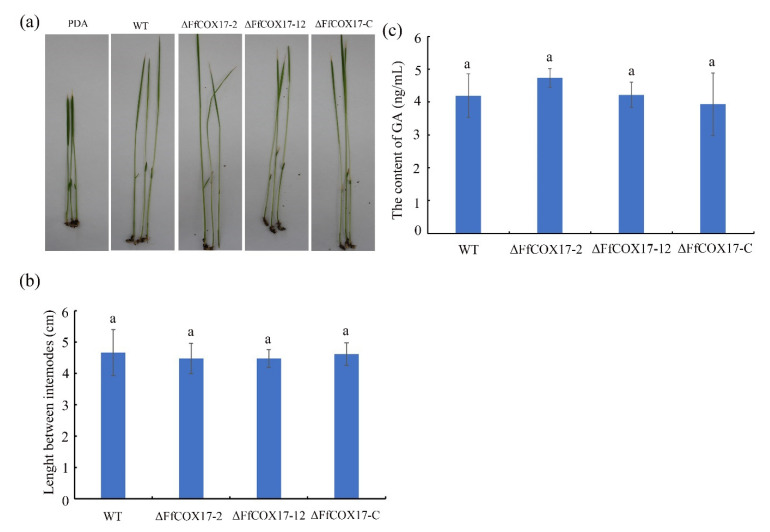
The effects of *FfCOX17* on pathogenicity of *Fusarium fujikuroi*. (**a**) Pathogenicity of wild-type, *ΔFfCOX17*-2, *ΔFfCOX17*-12 and complemented strain on rice seedlings. (**b**) Rice seedling lesion length of wild-type WT, *ΔFfCOX17*-2, *ΔFfCOX17*-12 and complemented strain. (**c**) The GA content of different strains. Values in each column with the same letter are not significantly different (Tukey; *p* = 0.05).

The rice seedlings infection assay was performed to assess the role of *FfCOX17* in the pathogenicity of *F. fujikuroi*. The *∆FfCOX17* mutants caused similar lesion lengths compared with the wild-type strain (Figure 5a,b, Table 1), which indicated that *FfCOX17* is not essential for plant infection by *F. fujikuroi*. To further confirm whether the pathogenicity is related to GA production, the GA content was measured using a GA ELISA detection kit. The GA content of the wild-type strain is 4.95 ng/mL, and the GA content of *∆FfCOX17* mutants is 4.73 ng/mL and 4.22 ng/mL, respectively, suggesting that the GA content in *∆FfCOX17* mutants was similar to the wild-type strain (Figure 5c). 

### 2.6. Sensitivity of the ∆FfCOX17 to Different Stresses

**Figure 6:**
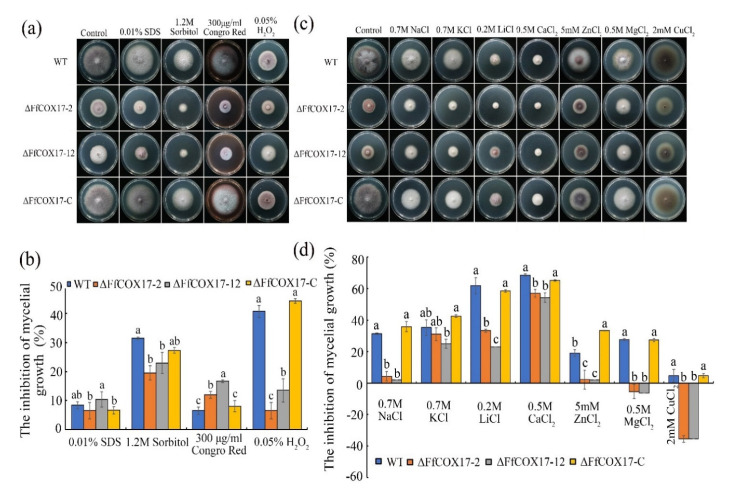
Sensitivity of the *ΔFfCOX17* to different stresses and metal cation; (**a**) Growth phenotype of wild-type WT, *ΔFfCOX17*-2, *ΔFfCOX17*-12 and complement strain on PDA amended with 0.01% SDS, 1.2 M Sorbitol, 300 μg/mL Congo Red, and 0.05% H_2_O_2_ after 7 days of incubation at 25 °C. (**b**) The inhibition of mycelial growth of wild-type WT, *ΔFfCOX17*-2, *ΔFfCOX17*-12 and complement strain to *ΔFfCOX17* to 0.01% SDS, 1.2 M Sorbitol, 300 μg/mL Congo Red, and 0.05% H_2_O_2_. (**c**) The colony morphology of wild-type WT, *ΔFfCOX17*-2, *ΔFfCOX17*-12 and complement strain on PDA containing 0.7 M NaCl, 0.7 M KCl, 0.2 M LiCl, 0.5 M CaCl_2_, 5 mM ZnCl_2_, 0.5 M MgCl_2_ or 2 mM CuCl_2_ after 7 days of incubation at 25 °C. (**d**) The inhibition of mycelial growth of the *ΔFfCOX17* to metal cation 0.7 M NaCl, 0.7 M KCl, 0.2 M LiCl, 0.5 M CaCl_2_, 5 mM ZnCl_2_, 0.5 M MgCl_2_ or 2 mM CuCl_2_. Values in each column with the same letter are not significantly different (Tukey; *p* = 0.05).

**Figure 7:**
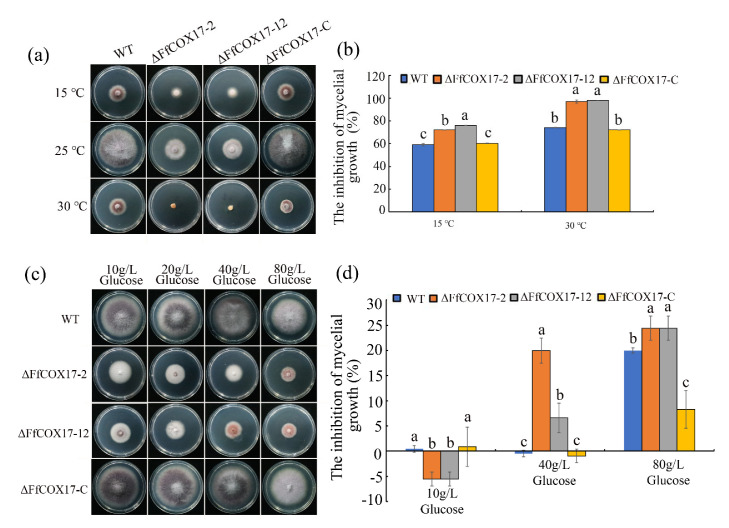
Sensitivity of the *ΔFfCOX17* to heat shock and different concentration glucose; (**a**) The colony morphology of wild-type, *ΔFfCOX17*-2, *ΔFfCOX17*-12 and complement strain to different temperatures (15 °C and 30 °C). (**b**) The inhibition of mycelial growth of wild-type WT, *ΔFfCOX17*-2, *ΔFfCOX17*-12, and complement strain at different temperatures (15 °C and 30 °C). (**c**) Sensitivity of wild-type WT, *ΔFfCOX17*-2, *ΔFfCOX17*-12 and complement strain to different concentration glucose. (**d**) The inhibition of mycelial growth of wild-type, *ΔFfCOX17*-2, *ΔFfCOX17*-12 and complement strain to 10 g/L Glucose, 40 g/L Glucose, and 80 g/L Glucose. Values in each column with the same letter are not significantly different (Tukey; *p* = 0.05).

Environmental stress factors play an important role in the process of pathogen infection. As shown in Figure 6, *∆FfCOX17* displayed decreased sensitivity to 1.2 M Sorbitol, 0.05% H_2_O_2_, 2 mM CuCl_2_, 0.7 M NaCl, 0.2 M LiCl, 0.5 M CaCl_2_, 5 mM ZnCl_2_ and 0.5 M MgCl_2,_ but significantly increased sensitivity to 300 μg/mL Congo Red (Figure 6a–d). The sensitivity of *ΔFfCOX17* mutants to heat shock was also detected at different temperatures, and the results indicated that *ΔFfCOX17* displayed increased sensitivity at 15 °C and 30 °C (Figure 7a,b). *ΔFfCOX17* exhibited increased sensitivity to 40 g/L glucose, 80 g/L glucose, and decreased sensitivity to 10 g/L glucose (Figure 7c,d). All growth defects of *ΔFfCOX17* mutants in response to different stresses were restored by complemented strain *ΔFfCOX17*-C. These data suggested that *FfCOX17* is associated with membrane permeability, cell wall integrity, and sensitivity to environmental factors.

### 2.7. FfCOX17 Regulates the Sensitivity to Different Fungicides

**Figure 8:**
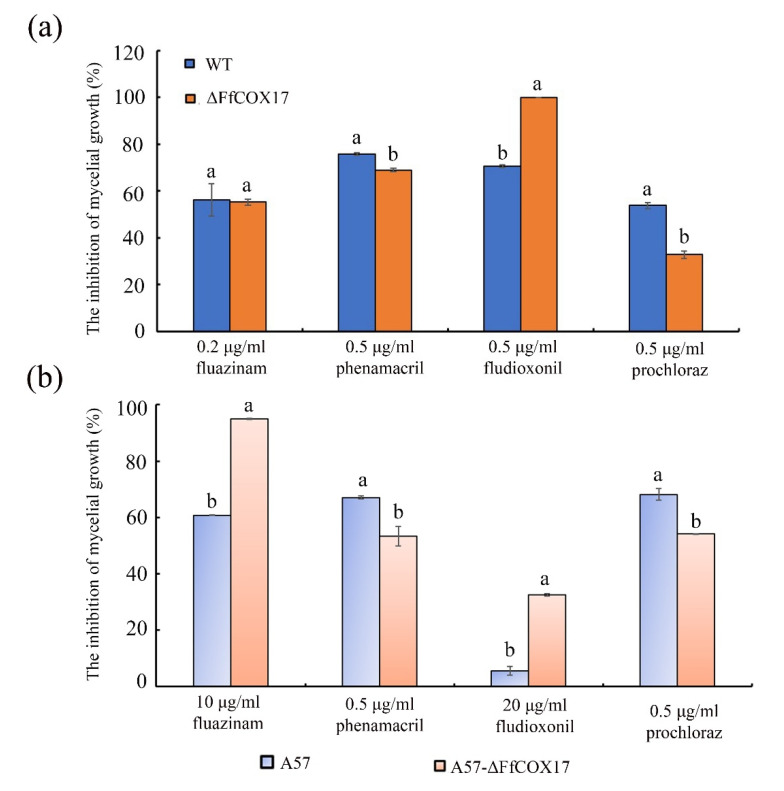
The sensitivity of *ΔFfCOX17* to different fungicides. (**a**) The inhibition of mycelial growth of wild-type WT and *ΔFfCOX17* mutants to 0.2 μg/mL fluazinam, 0.5 μg/mL phenamacril, 0.5 μg/mL fludioxonil and 0.5 μg/mL prochloraz. (**b**) The sensitivity of fluazinam-resistant strain A57 and A57-*ΔFfCOX17* mutant to 10 μg/mL fluazinam, 0.5 μg/mL phenamacril, 20 μg/mL fludioxonil and 0.5 μg/mL prochloraz. Values in each column with the same letter are not significantly different (Tukey; *p* = 0.05).

The sensitivity of *F. fujikuroi* wild-type strain, fluazinam resistant strain A57, and the *ΔFfCOX17* mutants in WT and A57 backgrounds to different fungicides were determined. Under the wild-type strain background, the inhibition rate of WT by 0.5 μg/mL fludioxonil was 70.66% but increased to 100% in the *ΔFfCOX17* strains. However, the inhibition rate by 0.5 μg/mL prochloraz was 53.72% in the wild-type strain and decreased to 32.74% in *ΔFfCOX17* (Figure 8a). Under the fluazinam resistant strain A57 background, the inhibition rate of A57 mycelium growth by 10 μg/mL fluazinam was 60.80% but increased to 95% in A57-*ΔFfCOX17*. Similarly, the inhibition rate of A57 by 20 μg/mL fludioxonil was 5.67% and increased to 32.50% in A57-*ΔFfCOX17*. However, the inhibition rate of A57-*ΔFfCOX17* by 0.5 μg/mL phenamacril and 0.5 μg/mL prochloraz decreased compared to that of A57 (Figure 8b). 

### 2.8. Subcellular Localization of GFP-FfCOX17 Fusion Protein

**Figure 9:**
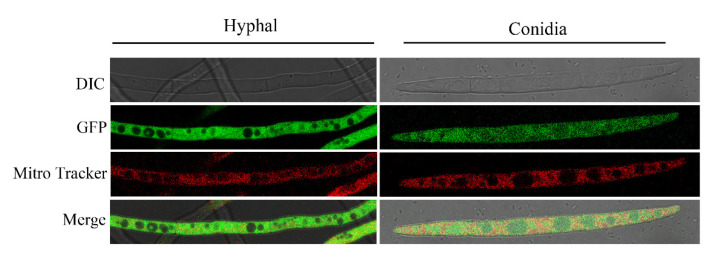
Subcellular localization of *FfCOX17* in mycelial and conidia.

To determine the subcellular localization of *FfCOX17*, the GFP-*FfCOX17* strain was generated. Figure 9 showed that the green fluorescence signals were visualized in the cytoplasm and mitochondria in mycelium and conidia as GFP signals and red signals of mitochondrial Mito marker partially overlapped, indicating that the *FfCOX17* was localized in mitochondria and cytoplasm.

## 3. Discussion

In this study, the copper chaperone protein *FfCOX17* was identified in *F. fujikuroi*. The growth rate of *ΔFfCOX17* on the different mediums was significantly lower than that of the wild-type strain. Beyond that, the conidia production was significantly decreased, and the germ tube length of *ΔFfCOX17* after germination for 12 h was shorter than wild-type strains, which indicates that *FfCOX17* regulates the vegetative growth and asexual reproduction of *F. fujikuroi*. In *S. cerevisiae* and *Arabidopsis*, the copper chaperone protein COX17 is essential to cell growth and stress response [22,25]. COX17 is involved in CcO assembly in yeast and mammalian cells [26]. Previous studies proved that the deletion or silence of the *AtCOX17* gene could lead to the growth defect in *Arabidopsis* [22]. In yeast, COX17 is located in mitochondria and affects cell respiration [26]. COX17 knockout could cause cell respiratory defects in mice [27]. In *A. nidulans*, the COX17 deletion mutant significantly reduced the mycelial growth rate and formed a small non-reproducible aconidial colony, indicating that COX17 is a necessary gene in *A. nidulans* [24]. The above results indicated that the COX17 homologous gene has functional characteristics.

Our results showed that the red pigment of the *∆FfCOX17* decreased significantly in the ICI medium. Polyketide synthase gene BIK has been proved to be a factor in the formation of red pigment of mycelial and a total of six genes were involved in BIK synthesis in *F. fujikuroi* [28]. Interestingly, the expression levels of the six BIK genes were significantly decreased in *∆FfCOX17* relative to the wild-type strain, suggesting that *FfCOX17* could regulate the pigment formation of *F. fujikuroi* by reducing the expression levels of BIK cluster genes. Filamentous fungi produce a variety of secondary metabolites and play different roles in cell physiological and biochemical processes [29,30,31]. Fumonisins could cause several animal diseases and are associated with some human diseases, which can inhibit ceramide synthase [10]. Previous studies indicated that deletion of *FvSEC4*, *FvDIM5*, and *FvCPSA* led to increased production of fumonisin [7,16,32]. In this study, *∆FfCOX17* was found to increase FUM content compared to the wide-type strain. Furthermore, the expression level of the *FfFUM2* gene was significantly increased in *∆FfCOX17*, which suggested that *FfCOX17* regulates the FUM content by increasing the expression levels of the *FfFUM2* gene. RBD caused by *F. fujikuroi* results in abnormal elongation of plants, reduction of tillers, sterility, or empty grains, and most of these symptoms are caused by the production of plant hormone GA [6]. The content of GA and pathogenicity in the *∆FfCOX17* mutant strain did not change compared with the control strain.

In yeast, COX17 can transport copper between the mitochondrial inner membrane and cytoplasm [33]. Silencing of the *AtCOX17* gene resulted in decreased response to salt stress, and *AtCOX17* is necessary for stress response gene expression levels in *Arabidopsis* [22]. In yeast and mammals, COX17 protein contains six conserved cysteine residues, which are involved in redox reaction and metal binding and transport, respectively [26]. Mammalian COX17 exists in three oxidation states, COX17_0S-S_, COX17_2S-S_, and COX17_3S-S_, respectively. COX17_0S-S_ combines with Cu^+^; COX17_2S-S_ binds to Cu^+^ or Zn^2+^; COX17_3S-S_ does not bind to any metal [34,35]. The *∆FfCOX17* mutants showed decreased sensitivity to metal ion such as 5 mM ZnCl_2_, 2 mM CuCl_2_, 0.5 M MgCl_2_ and 0.7 M NaCl. In addition, the *∆FfCOX17* mutants displayed decreased sensitivity to oxidative stress factors such as 0.05% H_2_O_2_ and increased sensitivity to cell wall-damaging agents 300 μg/mL Congo Red, which indicated the cell wall integrity of *∆FfCOX17* was destroyed. In *B. cinerea* and *S. sclerotiorum*, there was positive cross-resistance between fludioxonil and fluazinam [36,37]. Fludioxonil can induce glycerol biosynthesis and interfere with osmotic signal transduction in *C. albicans* [38]. Deletion of *FfCOX17* increased the sensitivity of *F. fujikuroi* to fluazinam and fludioxonil, but significantly decreased sensitivity to phanamacril and procloraz. *∆FfCOX17* displayed decreased sensitivity to osmotic stress factor 0.7M NaCl and 1.2M Sorbitol, which may be due to the osmotic pathway being disturbed, increasing the sensitivity of *F. fujikuroi* to fungicides fluazinam and fludioxonil. The results indicated that fluazinam or fludioxonil could be combined with phenamacril or procloraz as an effective fungicide strategy to control RBD. 

## 4. Conclusions

In summary, we identified the copper chaperone protein *FfCOX17* in *F. fujikuroi*, and a localization study found that *FfCOX17* is located in mitochondria and cytoplasm. *FfCOX17* deletion mutants showed a decrease in vegetative growth and asexual reproduction, sensitivity to oxidative stress, osmotic stress, and increased sensitivity to cell wall stress, heat shock stress, and high concentration glucose. In addition, *∆FfCOX17* also showed increased sensitivity to fungicide fluazinam and fludioxonil and decreased sensitivity to phenamacril and prochloraz. Interestingly, the transcriptional level of the *FfFUM2* gene was significantly upregulated in *∆FfCOX17*, and the fumonisin production in the *∆FfCOX17* mutants was significantly increased, but the *FfCOX17* is not related to virulence. Future studies will focus on analyzing the molecular mechanism of *FfCOX17* negatively regulating FUM production.

## 5. Materials and Methods

### 5.1. Fungal Strains, Media, and Culture Conditions

The wild-type strain WT of *F. fujikuroi* was collected from rice fields in Jiangsu Province of China in 2019. Briefly, the disease sample of RBD was randomly collected and disinfected in a sodium hypochlorite solution (5% available chlorine) for 45 s. Then, they were rinsed thrice with sterile water and dried. The disinfested disease sample was placed on a potato dextrose agar (PDA) plate containing 100 μg/mL streptomycin sulfate. The PDA plate was incubated at 25 °C for 5 days. Purified strains of *F. fujikuroi* were obtained by the single spore method. The strain was maintained on PDA slants at 4 °C. Finally, the wild-type strain A was verified by ITS sequencing and morphology. WT strain and fluazinam-resistant strain A57 (induced in the laboratory) were used as parental strains to obtain the deletion mutants of *FfCOX17*, and the complementary strain was obtained from the *ΔFfCOX17* mutant.

PDA contains 200 g of potato, 20 g glucose, 16 g of agar, and 1 L of water. Complete medium (CM) was made from 50 mL nitrate salts (NaNO_3_ 120 g, KCl 10.4 g, MgSO_4_·7H_2_O 10.4 g, KH_2_PO_4_ 30.4 g per liter of distilled water), 1 mL trace element (ddH_2_O 80 mL, ZnSO_4_·7H_2_O 2.2 g, H_3_BO_3_ 1.1 g, MnCl_2_·4H_2_O 0.5 g, FeSO_4_·7H_2_O 0.5 g, CoCl_2_·6H_2_O 0.17 g, CuSO_4_·5H_2_O 0.16 g, Na_2_MoO_4_·2H_2_O 0.15 g, Na_4_EDTA 5 g, pH 6.5 per liter of distilled water), 10 g glucose, 2 g peptone, 1 g yeast extract, 1 g casein hydrolysate, 1 mL 1% thiamine, 50 μL 0.05% biotin solution, 15 g agar, pH 6.5 per liter of distilled water. Minimal medium (MM) consisted of 50 mL nitrate salts, 1 mL trace element, 10 g glucose, 1 mL 1% thiamine, 50 μL 0.05% biotin solution, 18 g agar, pH6.5 per liter of distilled water. V8 medium was made from 200 mL V8 juice, 2 g CaCO_3_, and 20 g agar per liter of distilled water. YEPD medium consisted of 20 g glucose, 10 g peptone, and 3 g yeast extract per liter of distilled water. Spore production was measured after incubation for 7 days in carboxymethylcellulose (CMC) liquid medium (15 g carboxymethyl cellulose, 0.5 g NH_4_NO_3_, 0.5 g KH_2_PO_4_, 0.25 g FeSO_4_·7H_2_O, 0.5 g yeast extract per liter of distilled water).

### 5.2. Construction of Vectors, Fungal Transformation and Generation of Gene Deletion

To investigate the functions of *FfCOX17* in *F. fujikuroi*, we generated two independent *FfCOX17* deletion mutants. A gene replacement carrier *ΔFfCOX17* carrying the hygromycin resistance gene (*hph*) and herpes simplex virus thymidine kinase gene (*F_2_du*), an upstream fragment (5’) of *FfCOX17*, and downstream fragment (3’) of *FfCOX17* were amplified from the genome DNA of WT with primers listed in Table S1, the 3490 bp hph-hsv (hph and F_2_du) fragment was amplified from the hph-hsv plasmid DNA with primers hph-hsv-UF/hph-hsv-UR, the three fragments were fused by single point PCR split-marker approach [29]. The fusion product was amplified using primers *FfCOX17*-UF/*FfCOX17*-DR and added to the protoplast of the wild-type strain. We used a 50 μL polymerase chain reaction (PCR) system including 25 μL LAmp Master Mix (Vazyme Biotech Co., Ltd, Nanjing), 2 μL forward primer, 2 μL reverse primer, 1 μL total DNA, and 20 μL water. Reaction procedure: predenaturation at 94 °C for 5 min; 35 cycles: denaturation at 94 °C for 30 s; annealing at 56 °C for 30 s, extension at 72 °C for 30 s/kb; thoroughly extend at 72 °C for 7 min. The protoplasts were prepared from *F. fujikuroi* hyphae according to previous research [39]. All of the transformants were verified by PCR with different primers (Table S1) and further verified by Southern blotting. 

To construct the *FfCOX17*-GFP fusion vector, the GFP fusion fragment of *FfCOX17* was amplified using primers *FfCOX17*-RP27-GFP-F/*FfCOX17*-GFP-R and cloned into Pyf11 plasmid vector (XhoI digestion) using 2MultiF Seamless Assembly Mix (ABclonal Technology Co., Ltd, Wuhan), and then transferred to *E. coli* (DH5α) for amplification. The *FfCOX17*-GFP fusion vector was added to the protoplast of the wild-type strain to obtain the *FfCOX17*-GFP strain. The fluorescence signal (wavelength range of green fluorescence is 460 nm~550 nm) was taken under a confocal microscope (Leica TCS SP8). 

### 5.3. Test for Vegetative Growth and Asexual Reproduction

The wild-type strain, deletion mutants *ΔFfCOX17* (*ΔFfCOX17*-2 and *ΔFfCOX17*-12) and complement strain *ΔFfCOX17*-C were cultured on a PDA medium for 6 days, a 5 mm plug was cut from the colony margin and placed on PDA, V8, CM, and MM medium at 25 °C for 7 days. Each treatment had three replicates, and the diameter of each plate was measured after seven days of culture.

For asexual reproduction, three mycelial plugs (diameter: 5 mm) of different strains were taken from the colony’s edge which was cultured on a PDA medium for 6 days and then transferred into a 250 mL flask containing 100 mL CMC liquid medium. All of the flasks were shaken at 25 °C, 175 rpm for 7 days. The number of conidia in the CMC liquid medium of each strain was determined by hemocytometer. The experiments were performed three times with three replicates for each treatment.

### 5.4. Pathogenicity Assays

The seeds of rice variety Ninggeng 7 were prepared, and the surface was disinfected. Briefly, the peeled rice seeds were sterilized with 75% ethanol for one minute, rinsed with sterile water three times, soaked with sodium hypochlorite (4% available chlorine) for 10 min, and rinsed with sterile water three times. The sterilized seeds were transferred into water agar plates (15 g/L agar) cultured for 4 days at low temperature (4 °C), and then transferred to a 28 °C light incubator (alternating light and dark for 12 h) for germination for 3 days. Place the germinated seeds in 3 × 20 cm test tubes (filled with 25% vermiculite), the mycelial plug (5 mm in diameter) of different strains cultured on a PDA medium for 6 days was transferred into the test tubes and add 3 mL Gamborg B5 solution (3.16 g/L) (Duchefa Biochemie B.V. Holland) to each test tube. The mycelial plug of the PDA plate was added as a control. The test tubes were placed in a light incubator at 28 °C for 12 h-light and 12 h-dark cycle conditions for 7 days. Finally, the length of the seedling was measured from the stem base to the second root nodule.

### 5.5. FUM and GA Content Assay

To determine the content of FUM and GA, three mycelial plugs were cut from the colony margin of cultured on a PDA medium for 6 days and transferred into the conical flask containing 100 mL ICI liquid medium (containing 6 mM Gln) [6], the flasks were shaken at 28 °C, 175 rpm for one week in darkness. After 7 days of culture, the culture solution was collected for the determination of FUM or GA content. The 50 μL sample solution and standards were added to the microwell plate, respectively, joining 50 μL anti-FUM antibody conjugate (or 50 μL anti-GA antibody conjugate), gently mixing for a few seconds, 37 °C warm bath 30 min, wash 5 times, add the chromogen solution at 37 °C and incubate it again for 10 min, add the stop solution, detect the absorbance at 450 nm, and the FUM or GA content was calculated according to the standard curve. 

### 5.6. Quantitative RT-PCR (qPCR)

For gene expression, three mycelial plugs were cut from the colony margin of cultured on a PDA medium for 6 days and placed in the conical flask containing 100 mL ICI liquid medium (containing 6 mM Gln), the flasks were shaken at 28 °C, 175 rpm for 48 h in darkness. RNA samples were isolated from 48 h hyphal with RNAsimple Total RNA Kit (Tiangen Biotech CO., Ltd, Beijing, China). The first-strand cDNA was synthesized by HiScript II RT SuperMix for qPCR (+gDNA wiper) (Vazyme Biotech Co., Ltd, Nanjing, China). qPCR was performed with ChamQ SYBR qPCR Master Mix (Vazyme Biotech Co., Ltd, Nanjing, China) by CFX Connect Real-Time System (Bio-Rad, USA) [40]. To quantify mRNA levels of *FfFUM* (FUM2, FFUJ_09248), *FfBIK1* (FFUJ_06742), *FfBIK2* (FFUJ_06743), *FfBIK3* (FFUJ_06744), *FfBIK4* (FFUJ_06745), *FfBIK5* (FFUJ_06746), and *FfBIK6* (FFUJ_06747), we used primers FfFUM2-DL-F/FfFUM2-DL-R, FfBIK1-DL-F/FfBIK1-DL-R, FfBIK2-DL-F/FfBIK2-DL-R, FfBIK3-DL-F/FfBIK3-DL-R, FfBIK4-DL-F/FfBIK4-DL-R, FfBIK5-DL-F/FfBIK5-DL-R, and FfBIK6-DL-F/FfBIK6-DL-R, respectively (Table S1). The actin gene (FFUJ_05652) was used as an internal reference gene. The relative expression level of different genes was calculated according to the reference gene using the 2^−ΔΔCt^ method.

### 5.7. Sensitivity of the ΔFfCOX17 Mutants to Different Stress

To determine the sensitivity of *ΔFfCOX17* to different stresses, the mycelia plug (diameter 5 mm) was taken from the edge of the colony, which was cultured on a PDA medium for 6 days and placed on the PDA plate amended with different metal cation (0.7 M NaCl, 0.7 M KCl, 0.2 M LiCl, 0.5 M CaCl_2_, 5 mM ZnCl_2_, 0.5 M MgCl_2_ or 2 mM CuCl_2_), 300 μg/mL Congo Red (cell wall stress factor), 0.01% SDS (cell membrane stress factor), or 0.05% H_2_O_2_ (oxidative stress). In addition, some mycelial plugs were transferred into the PDA plate containing 10 g/L glucose, 20 g/L glucose, 40 g/L glucose, and 80 g/L glucose. All PDA plates were cultured in the incubator under dark conditions for 7 days. For the sensitivity of *ΔFfCOX17* to heat shock, the mycelia plugs were placed on the PDA plate and incubated at 15 °C, 25 °C, or 30 °C incubators for 7 days in darkness. The colony diameter of each treatment was measured and the inhibition rate was calculated using the formula: inhibition rate = (the diameter of the treatment − the diameter of control)/(the diameter of control − 0.5) × 100. Each treatment had three repetitions, and the experiment was repeated three times independently.

### 5.8. Determination of the Sensitivity of F. fujikuroi to Different Fungicides

The wild-type strain, *ΔFfCOX17* mutants, and complemented strain *ΔFfCOX17*-C were used to determine the sensitivity of *F. fujikuroi* to different fungicides. A 5 mm diameter mycelial plug was cut from the edge of the 6 days PDA colony and transferred onto the PDA plates amended with 0.2 μg/mL fluazinam, 0.5 μg/mL phenamacril, 0.5 μg/mL fludioxonil, and 0.5 μg/mL prochloraz (sensitive strain and *ΔFfCOX17* mutant), or 10 μg/mL fluazinam, 0.5 μg/mL phenamacril, 20 μg/mL fludioxonil and 0.5 μg/mL prochloraz (fluazinam-resistant strain and *ΔFfCOX17* mutant). The colony diameter was measured after it was incubated at 25 °C for 7 days in darkness and used to calculate the mycelial growth inhibition.

### 5.9. Statistical Analysis

The experimental data were analyzed using SPSS statistical software. Statistical analysis was performed using one-way variance (ANOVA), followed by the Tukey multiple comparison test. The level of significance was set at *p* < 0.05. All of the experiments were performed three times with three replicates for each treatment. Adobe Photoshop CS5 was used to draw pictures, and Excel, PowerPoint, and other office software were used to sort out relevant data and draw basic charts.

## Figures and Tables

**Table 1:** Phenotypes of the *FfCOX17* deletion mutant (*ΔFfCOX17*-2 and *ΔFfCOX17*-12), the parental strain (WT), and the complemented strain (*ΔFfCOX17*-C) in terms of growth, conidiation, and virulence.

Strain	Vegetative Growth (cm)				Conidiation (×10^6^/mL)	Pathogenicity
	PDA	V8	CM	MM		Lesion Length (cm)
WT	5.13 ± 0.05 ^b^	4.80 ^a^	5.0 ± 0.35 ^a^	5.50 ^a^	8.50 ± 0.35 ^a^	4.67 ± 0.73 ^a^
*ΔFfCOX17*-2	3.15 ± 0.06 ^c^	2.35 ± 0.06 ^b^	3.35 ± 0.06 ^b^	3.30 ^b^	0.28 ± 0.10 ^b^	4.48 ± 0.48 ^a^
*ΔFfCOX17*-12	3.10 ^c^	2.35 ± 0.06 ^b^	3.30 ^b^	3.40 ^b^	0.27 ± 0.06 ^b^	4.48 ± 0.28 ^a^
*ΔFfCOX17*-C	5.55 ± 0.06 ^a^	4.80 ^a^	4.75 ± 0.06 ^a^	5.13 ± 0.05 ^a^	7.94 ± 0.44 ^a^	4.61 ± 0.36 ^a^

Note: Values in each column with the same letter are not significantly different (Tukey; *p* = 0.05). Abbreviations: PDA, potato dextrose agar; V8, V8 juice agar; CM, complete medium; MM, minimal medium.

## Data Availability

Strains and plasmids are available upon request. This research contains the original method of this study, and further inquiries can be directly contacted by the corresponding authors.

## Supplementary Materials

The following are available online at: https://www.mdpi.com/article/10.3390/toxins14070427/s1, Table S1: Primers used in this study.
