# Supplementary material for: FfCOX17 is Involved in Fumonisins Production, Growth, Asexual Reproduction, and Fungicide Sensitivity in Fusarium fujikuroi
Source: Toxins (Basel). 2022 Jun 22;14(7):427. doi: 10.3390/toxins14070427 (PMC9319711; doi:10.3390/toxins14070427)
Supplement: Supplementary file 1 [file toxins-14-00427-s001.zip › toxins-1739174-supplementary.pdf]

Supplementary Materials: FfCOX17 is involved in fumonisins production, growth, and fungicide sensitivity in *Fusarium fujikuroi*

Xuewei Mao, Zhiwen Wu, Furong Chen, Mingguo Zhou and Yiping Hou

Table S1. Primers used in this study.

Primers	Sequence (5'-3')	Application
FfCOX17-UF	TTCATATCCCATCCGTTAA	amplification of the left junction of FfCOX17 deletion mutant
FfCOX17-UR	GCTCCTTCAATATCATCTTCTGTCGTGTCTGATTTTAA	
FfCOX17-DF	AGACAATACCGGAAGGAACAAGGTTCACAACTTCA	amplification of the right junction of FfCOX17 deletion mutant
FfCOX17-DR	AACAACTCCTTTCGGTCTA	
FfCOX17-inF	GACGCAGCTCAGACAACA	verify the target gene of FfCOX17 deletion mutant
FfCOX17-inR	GCTTAGCCTTCTCATCCTT	
FfCOX17-yzsF	GTGGGCACTCCCTCGTCCTT	verify the left junction of FfCOX17 deletion mutant
FfCOX17-yzsR	GTCCTCGTTCCTGTCTGCTAAT	
FfCOX17-yzxF	ATACCACCTCAAATCACCCA	verify the right junction of FfCOX17 deletion mutant
FfCOX17-yzxR	TGACGGAGCTACGACACC	
Hph-hsv-F	ACAGAAGATGATATTGAAGGAGC	amplification of the fragment of hph-hsv
Hph-hsv-R	GTTCCTTCCGGTATTGTCTC	
FfFUM2-DL-F	TGGGTCTAGTGAGGAGCTTAC	determine the relative expression level of the FUM gene
FfFUM2-DL-R	CGATGGGAACAGGTGGATAAC	
ACTIN-DL-F	CATTCTGCGGAGGAAACCTATC	reference gene qPCR primers
ACTIN-DL-R	CTCCTTGTCAACCTCTTCCATAAA	
FfBik1-DL-F	GAGCAGGTTCATGGTGATAAGA	determine the relative expression level of the BIK1 gene
FfBik1-DL-R	AGAGTCGAAGGAGCCATAGT	
FfBik2-DL-F	CGCACAGCCAGCTACAAC	determine the relative expression level of the BIK2 gene
FfBik2-DL-R	CCATAAAGGCACGCAAAA	
FfBik3-DL-F	CACTGTTGAGTCTATGGGTCAG	determine the relative expression level of the BIK3 gene
FfBik3-DL-R	CCACATCACGAGATCGAAGAA	
FfBik4-DL-F	TTATCAACCGGGAGCTTGTC	determine the relative expression level of the BIK4 gene
FfBik4-DL-R	AGAGACTTGGCTACAACATCAG	
FfBik5-DL-F	CCAACACCAGCGTACTCTAATAA	determine the relative expression level of the BIK5 gene
FfBik5-DL-R	CATCTGACTGACCACTGCTATC	
FfBik6-DL-F	TCCAATCGTCCCTCCAGTAT	determine the relative expression level of the BIK6 gene
FfBik6-DL-R	CATACATGGGCCAGACAAAGA	
COX17-RP27-GFP-F	CAGATCTTGGCTTTCGTAGGAACCCAATCTTCAATGGACGCAGCTCAGACAACAA	amplification of the fragment of FfCOX17-GFP
COX17- GFP-R	CACCACCCCGGTGAACAGCTCCTCGCCCTTGCTCACCACCTGGTATCCAAATCC	
